# Antagonist development for the CMG2 protein

**DOI:** 10.1063/4.0001127

**Published:** 2025-10-27

**Authors:** Prasadika Samarawickrama Hetti Arachchige, Fang Fang, James David Moody, Ken Christensen

**Affiliations:** Brigham Young University, Provo, Utah, USA

## Abstract

Capillary morphogenesis gene 2 (CMG2) plays an important role in angiogenesis and is an anthrax toxin receptor. It is involved in the cell adhesion and mobility of various cell types, such as epithelia and endothelia (Cryan, 2022). It is also involved in pathological processes such as eye disorders, cancer, arthritis, several rare genetic diseases, and psoriasis. Previous research has shown that inhibiting CMG2 yields powerful anti- angiogenic effects (Rogers, 2012). Utilizing derivatives of Anthrax Protective Antigen (PA), the treatment effectively suppresses angiogenesis in assays for growth-factor-induced corneal neovascularization. It also hinders endothelial cell chemotaxis towards various angiogenic growth factors, including VEGF, bFGF, and PDGF. Furthermore, it notably retards tumor growth across multiple tumor models (Becker, 2020). The antagonist development for the CMG2 protein will be able to provide antiangiogenic therapies with higher efficacy and lower toxicity. We have utilized a new protein scaffold of the Designed Ankyrin Repeat Protein (DARPin) to create a range of CMG2 antagonists with strong interactions with CMG2. We designed the amino acid sequence for CMG2 Inhibitory DARPins (CID) computationally through molecular docking and PyRosetta design. These initially designed CIDS exhibit remarkably low Kd values for CMG2, measuring around 30 pM. We were able to show that CID has an inhibitory action against CMG2 using a Chemotaxis assay and immunofluorescence. A second assay with a Mg-free vWa mutant does not show any inhibition by CIDs, revealing that CIDs bind to the vWa via the desired Mg-binding site. We were able to see the combination of 1TEL-vWA and CID proteins through size exclusion chromatography, but the protein complex didn’t provide crystals. So, we designed a double tether of 1TEL-vWA linked to CID and vWA alone linked to CID to see whether it would help the binding of CID and vWA. We obtained crystals for only 1TEL-vWA-CID, and those crystals were not large enough to diffract. We ran an SDS PAGE gel using shooted crystals and confirmed that they consist only of uncleaved protein. Thus, we are optimizing the conditions to obtain large and well-ordered crystals that can diffract with a good resolution. We aim to determine the atomic-level structures of the CID-vWa complex by obtaining a crystal structure for the complex. It will help us to understand the binding mode to the vWa, which will help in improving CID3’s properties.

